# Immune infiltrating cells in duodenal cancers

**DOI:** 10.1186/s12967-020-02508-4

**Published:** 2020-09-03

**Authors:** G. Donisi, G. Capretti, N. Cortese, A. Rigamonti, F. Gavazzi, G. Nappo, A. Pulvirenti, M. Sollai, P. Spaggiari, A. Zerbi, F. Marchesi

**Affiliations:** 1 Section of Pancreatic Surgery, Humanitas Clinical and Research Center-IRCCS, Via Manzoni 56, 20089 Rozzano, Milano Italy; 2 Department of Immunology and Inflammation, Humanitas Clinical and Research Center-IRCCS, Via Rita Levi Montalcini 4, Pieve Emanuele, 20090 Rozzano, Milano Italy; 3 Department of Medical Biotechnology and Translational Medicine, University of Milan, Milan, Italy; 4 Department of Pathology, Humanitas Clinical and Research Center-IRCCS, Rozzano, Italy; 5 Department of Biomedical Sciences, Humanitas University, Rozzano, Italy

**Keywords:** Duodenal cancer, Tumor infiltrating cells, Prognosis

## Abstract

**Background:**

Duodenal adenocarcinoma (DA) is a rare yet aggressive malignancy, with increasing incidence in the last decades. Its low frequency has hampered a thorough understanding of the pathogenesis of the disease and of its biology, limiting the identification of tailored therapeutic options. A large body of evidence has clearly shown the clinical relevance of immune cells in solid tumors, correlating immune features with post-surgical prognosis. The aim of this study was to analyze the immune contexture in a cohort of duodenal adenocarcinomas surgically resected at our Institution and define its correlation with clinical variables.

**Methods:**

Tissue slides from paraffin-embedded tumor specimens of 15 consecutive DA and 3 adenomas that underwent a pancreaticoduodenectomy in our center between 2010 to 2018 were immunohistochemically stained. The density (percentage of immune reactive area, IRA%) of immune markers CD45RO, CD8, CD20, IL-17, PD-1, CD68 was quantified by computer-assisted image analysis. Demographic, clinical, histopathological data were collected.

**Results:**

In our population, median IRA % (IQR) of immune subsets was respectively CD45RO-TILs 2.19 (2.14), CD8-TIL 0.42 (0.81), CD20-TILs 0.22 (0.51), CD20-TLT 2.84 (4.64), CD68-TAM 2.19 (1.56), IL17^+^ cells 0.39 (0.39), PD1-TILs 0.19 (0.41). The median follow-up was 47.5 (22.4–63.3) months. At statistical analysis, the density of CD8-TILs inversely correlated with lymph node ratio (p = 0.013), number of metastatic lymph nodes (p = 0.019), and was lower in N+ adenocarcinomas compared to N0 (1.07 vs 0.29; p = 0.093), albeit not significantly. Stratifying patients for the N status, the density of CD8-TILs decreased with the increasing of the N stage (p = 0.065) and was lower in patients who experienced recurrence and died for the disease (0.276 vs 0.641; p = 0.044). Notably, also CD68-TAM distribution was different in patients who had recurrence versus patients who did not (1.028 vs 2.276; p = 0.036).

**Conclusions:**

Immune cells showed variable expression in correlation with common prognostic factors, suggesting T cell infiltration may play a protective role towards lymphatic spread of disease and nodal metastatization. Furthermore, T cell density and macrophage infiltration were associated to a lower risk of recurrence and disease related death. A multicentric approach may be indicated to allow analysis of larger cohorts of patients, potentially increasing the power of our observations.

## Background

Within small bowel cancers (SBC), adenocarcinomas represent the 36.9% of the whole, the majority of them originating in the duodenum (49–58%) [1, 2]. Overall, duodenal adenocarcinomas (DA) represent less than 1% of all gastrointestinal cancers [3], yet they are aggressive malignancies, with increasing incidence in the last decades [1, 3, 4]. Surgical resection is the mainstay of treatment for local disease, but unfortunately, a large proportion of patients are not eligible, because the lesion is either locally advanced or metastatic [1, 3, 5]. In resected disease, the 5-year overall survival (OS) is 51.2%; the prognosis remains dismal, despite being more favorable than for other periampullary adenocarcinomas. Negative prognostic factors include presence of nodal and distant metastases, positive resection margins, lymphovascular and perineural involvement, high tumor (T) stage, large extent of nodal disease, and poorly differentiated histology, with lymph node metastases and lympn node ratio (LNR) having the highest prognostic yield [1, 5, 6].

The low frequency of duodenal cancers has hampered a refined characterization and a thorough understanding of the pathogenesis of the disease, since the few available studies, performed on mono-centric case series, often include patient cohorts spanning across several years and include both resected and unresectable lesions [7, 8]. The larger studies available either grouped duodenal adenocarcinoma with periampullary malignancies or with small bowel tumors, not being able to discriminate the peculiar features of DA [2, 9, 10]. Availability of limited information regarding the optimal surgical approach, the type and schedule of chemotherapy, the possibility to use immunotherapy has so far prevented from improvement in the clinical management of duodenal cancer patients. Moreover, patients with lesions at different stages are often grouped together, possibly hiding subgroups of patients who could benefit from adjuvant chemotherapy.

In consideration of these findings and of the failure to appropriately stratify patient prognosis with the currently available prognostic markers, the possibility to use immune-related characteristics as an additional factor in prognostic stratification algorithms has been recently investigated [11]. The tumor microenvironment (TME) is characterized by a variably large and heterogenous immune infiltrate, which has been variably associated to patient outcome across multiple cancer types [12, 13]. Critical features to be considered are the abundance of different populations, their localization in the core or at the invasive margin of the tumor and their spatial organization. Human colo-rectal cancer (CRC) has served as a prototype for studies aimed at assessing the clinical relevance of immune cells [14–17]. Based on this, an “immunoscore” inclusive of evaluation of CD3+ and CD8+ T cells infiltration at the tumor Core and invasive margin has been shown to reliably predict prognosis in CRC [18], and has been further validated in a multicenter study [19]. However, despite the close anatomic and histopathological similarities between the colonic and duodenal tissues, no studies have assessed the clinical relevance of immune cells in duodenal cancer. The lack of consensus on the clinical management and postsurgical therapeutic approaches for DA patients would benefit from the identification of variables contributing to better patient stratification.

## Methods

### Patients and study design

The cohort study included 18 patients aged older than 18 with histologically proven duodenal adenocarcinoma or adenoma, consecutively resected at the Humanitas Clinical and Research Center-IRCCS between 2010 and 2018. Histological diagnosis of ampullary neoplasm was an exclusion criterium. All patients included in the study had undergone either a pylorus-preserving pancreaticoduodenectomy by Longmire-Traverso or a Whipple procedure, according to the location of the lesion. Clinical features of the cohort are summarized in Table 1. Patients’ demographics, clinical, surgical and histopathological data were prospectively collected for following analyses and were retrospectively analyzed. Written informed consent was obtained from each patient included in the study. The study protocol was in accordance with the ethical guidelines established in the 1975 Declaration of Helsinki and compliant to the procedures of the local ethical committee of the institution. Tumors were staged according to TNM (Tumor-Nodes-Metastasis) staging system 8th ed. Investigators who performed the assessment of immune variables were blinded to the clinical data. The preoperative workup consisted of a variable combination of contrast-enhanced CT, EGDS, EUS, FNAB with histopathological examination. Clinical evaluation, biochemical analysis comprehensive of tumor markers, and whole-body CT scan, were carried out every 4 months, for the first 2 years and then every 6 months, according to the follow-up protocol of our institute. Time and location of recurrence, time and cause of death, and survival state were recorded for each patient.

**Table 1:** Demographic and clinico-pathological characteristics of included patients and pathological tumor characteristics

Patients and tumor characteristics	
Variable	Adenocarcinomas (n = 15)
Sex^a^	
Female	3 (20%)
Male	12 (80%)
Age at operation^b^	73 (65–78)
Clavien-Dindo ≥ IIIb^a^	5 (33%)
Post-operative mortality^a^	3 (20%)
DA on adenoma^a^	3 (20%)
Portion of duodenum involved^a^	
I	1 (7%)
II	13 (86%)
III	1 (7%)
IV	0 (0%)
TNM staging 7th edition	
T^a^	
1–2	2 (13%)
3–4	13 (87%)
N^a^	
0	7 (47%)
1	2 (13%)
2	6 (40%)
Stage grouping 7th edition^a^	
I	2 (13%)
II	5 (34%)
III	8 (53%)
Histological grade (G)^a^	
1–2	7 (46%)
3–4	8 (54%)
Resection status (R)^a^	
0, n (%)	14 (93%)
1, n (%)	1 (7%)
2, n (%)	0 (0%)
n° of retrieved lymph nodes^b^	23 (14–28)
n° of positive lymph nodes^b^	2 (0–3)
Lymph node ratio^b^	0.689 (0–0.115)
Perineural invasion^a^	5 (33%)
Lymphovascular invasion^a^	5 (33%)
Adjuvant chemotherapy^a^	6 (40%)
Adjuvant radiotherapy^a^	0 (0%)
Disease recurrence^a^	3 (20%)
Disease related death^a^	3 (20%)

Only adenocarcinoma patients have been included. ^a^ n (%); ^b^ median (IQR)

### Immunohistochemistry

2 μm-thick consecutive tissue sections were prepared from formalin-fixed and paraffin-embedded tissues, provided by the Pathology Department of the Humanitas Clinical and Research Center, and processed for immunohistochemistry. Briefly, after deparaffinization and rehydration, antigen retrieval was performed by heat treatment using EDTA buffer (0.25 mM, pH8, Dako) or citrate buffer (0.01 M, pH6, SIGMA-ALDRICH) in water bath at 98 °C for 20 min or pressure cooker. Endogenous peroxidases were blocked by incubation with 3% H_2_O_2_ for 15 min at room temperature, followed by incubation for 30 min with 2% BSA to block non-specific binding. The sections were then incubated with primary antibodies anti-human CD68 (Dako, KP-1 clone, diluted 1:1000), CD20 (Dako, L26 clone, diluted 1:200), CD8 (Dako, C8/144B clone, diluted 1:100), PD-1 (Abcam, NAT105 clone, diluted 1:50), CD45RO (Dako, UCHL1 clone, diluted 1:200), IL-17 (R&D, AF-317-NA, 1:500) for 1 h at room temperature, followed by incubation with the detection system MACH 1 (Biocare Medical) or Anti-Goat Polymer kit (Biocare Medical). Diaminobenzidine tetrahydrochloride (Biocare Medical) was used as chromogen. Nuclei were lightly counterstained with a freshly made hematoxylin solution (Dako). The sections were then washed in water, mounted and analysed under an optical microscopy.

### Image analysis

To obtain the density of immune cells, tissue slides were digitized after staining procedure, using a computer-aided slide scanner (Olympus VS120 DotSlide). An expert pathologist blinded to clinical data selected three non-contiguous, non-overlapping microscopic areas of stained slides in the invasive margin (IM) area, comprising approximately 50% of tumor and 50% of stromal tissue. For CD20-TLT analysis, 3 non-contiguous fields were chosen representing the entire CD20 positive area within the TLT, regardless of the location in the tumor or in the stroma. Both sampled microscopic area and light density were maintained fixed throughout the analysis. An image analysis software (Image Pro Premiere) was used to automatically determine the percentage of immune reactive area (IRA%) of the digitized images. The mean value, obtained from the three different microscopic areas, was calculated for each patient and used for subsequent analyses.

### Statistical analysis

Statistical computations were performed using the software IBM-SPSS (Chicago, USA), STATA 14.0 (StataCorp. 2015. College Station, TX) and the software GraphPad Prism 7 (GraphPad Software Inc., San Diego, USA). For image analyses, for each patient the mean value was calculated from three images. Correlation between IRA% values of immune cells were estimated by nonparametric Spearman Rank correlation coefficient test and linear regression analysis. Differences between immune variables in groups was estimated by non-parametric Mann–Whitney test. Kruskal–Wallis test was used to assess differences in median expression between more than two groups. For each test, only two-sided *P* values lower than 0.05 were considered statistically significant. The categorical variables were reported as a number and percentage, while continuous variables were reported as the median and interquartile range (IQR). Only patients with complete data were considered.

## Results

**Fig. 1:**
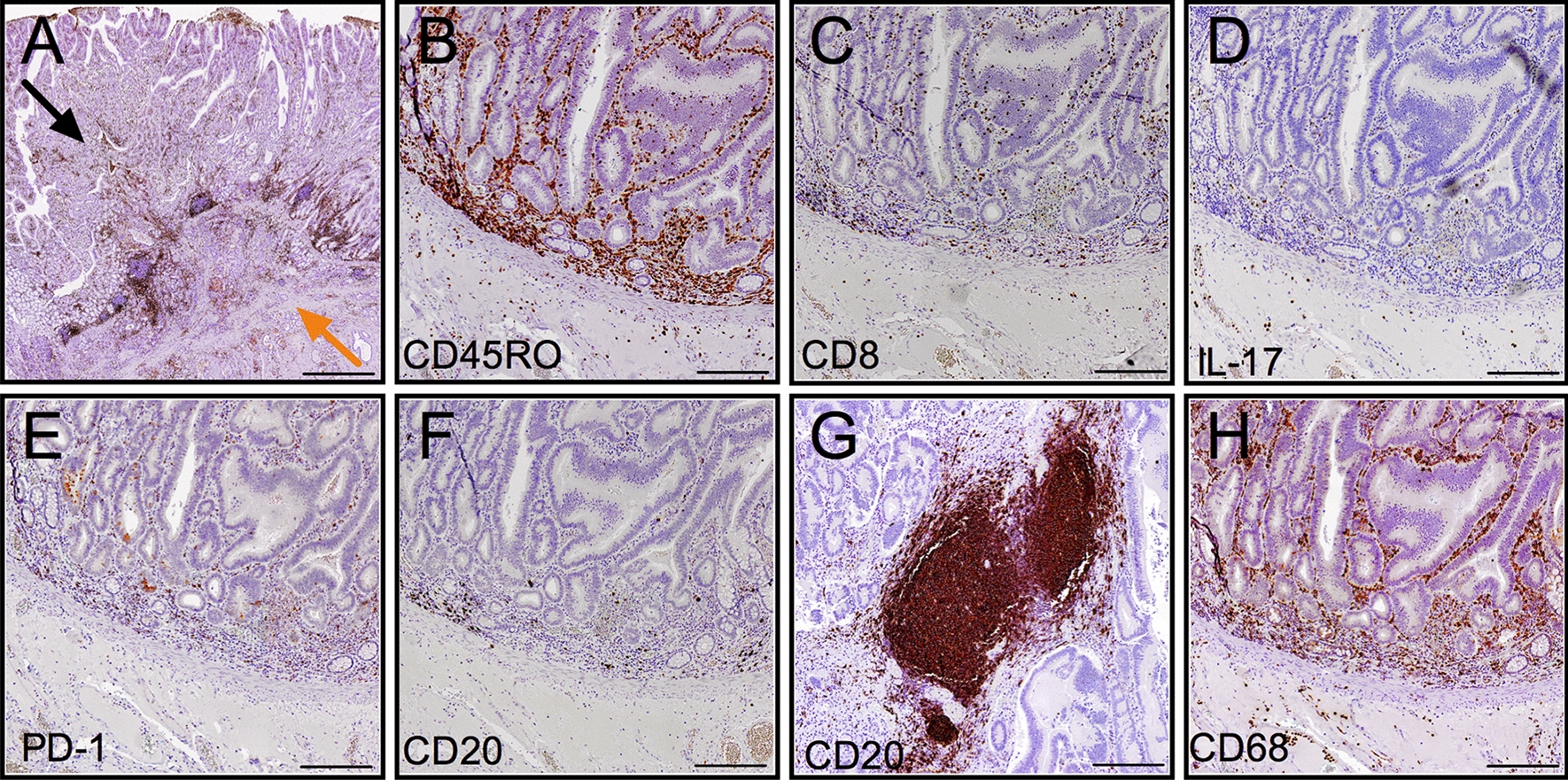
Immunohistochemical evaluation of immune cells in human duodenal cancer. **a** Representative picture of a human duodenal adenocarcinoma specimen; the invasive front (orange arrow) and tumor core regions (black arrow) are indicated. **b**–**h** Representative pictures of CD45RO-TILs (**b**), CD8-TILs (**c**), IL17+ cells (**d**), PD1-TILs (**e**), CD20-TILs (**f**), CD68-TAM (**h**) at the invasive margin and CD20-TLT (**g**) in the tumor stroma. Scale bars = 500 μm (**a**), 200 μm (**b**–**h**). TILs: tumor-infiltrating lymphocytes; TLT: tertiary lymphoid tissue; TAM: tumor-associated macrophages

Patient and tumor characteristics are listed in Table 1. The cohort included 15 duodenal cancers (DA) and 3 duodenal adenomas (Ad), from patients consecutively resected from 2010 to 2018 in our institution. Three among the adenocarcinomas progressed from an initial duodenal adenoma (DA on Ad). Histopathological evaluation of duodenal cancer specimens revealed an indeterminate histotype (Not Otherwise Specified, NOS) in the majority of cases, a mucinous component in 5 cases, one intestinal histotype and one adenosquamous. Most of the lesions were well circumscribed, often with a polypoid nature. An invasive front, where tumor cells intermingled with healthy tissue, was recognizable (Fig. 1a). The depth of invasion varied from perforation of the lamina propria to involvement of adjacent organs. The three adenoma specimens presented features similar to colon polyps, with delimitated borders and a clear base of implant, and had various grades of dysplasia from low to high. The median follow-up was 47.5 (22.4–63.3) months. At the time of analysis, we recorded 3 events of recurrence, respectively at 2.8 months, 4 and 17.8 months after surgery. Estimated Recurrence Free Survival (RFS) with (95% CI) was 74.2 months (56.4–92.0). The sites of recurrence were liver (1 patient), regional lymph nodes (1 patient) and combination of liver and interaortocaval lymph nodes (1 patient). All the three patients who relapsed experienced a disease related death respectively at 10.6 months, 12 and 33.8 months after surgery. Estimated disease-specific survival (DSS) with (95%CI) was 73.9 months (56.4-92.0).

### Immune cells infiltrating duodenal cancer specimens

**Fig. 2:**
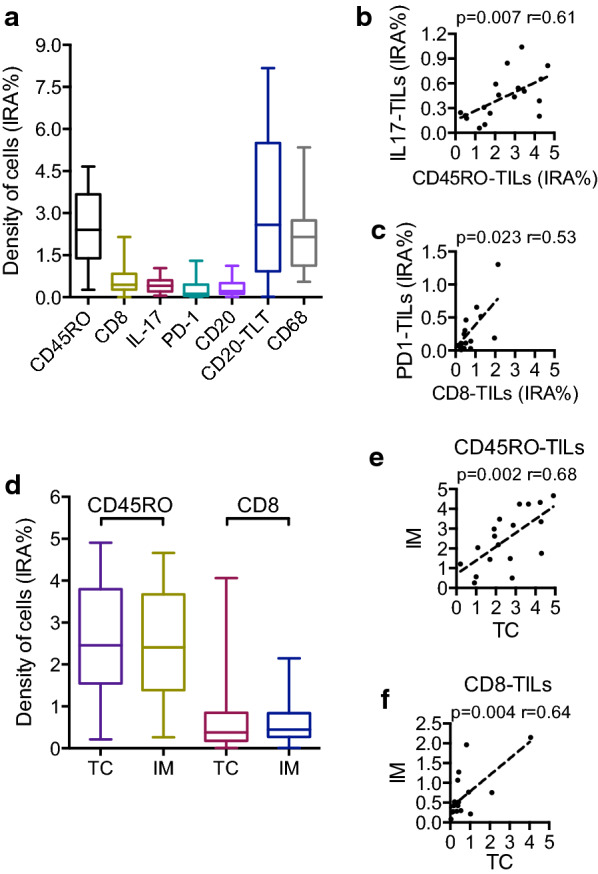
Distribution of immune cells in duodenal cancer specimens. **a** Density (IRA%, percentage of immunoreactive area) of immune cells at the invasive margin (n = 18). **b** Correlation between density (IRA%) of CD45RO-TILs and IL17^+^ cells at the invasive margin (n = 18; r = 0.61; p = 0.007 by Spearman analysis). **c** Correlation between density (IRA %) of CD8-TILs and PD1-TILs at the invasive margin (n = 18; r = 0.53; p = 0.023 by Spearman analysis). **d** Comparison between density (IRA%) of CD45RO-TILs and CD8-TILs in the tumor core (TC) and at the invasive margin (IM) (n = 18; p = ns by Mann–Whitney). **e**, **f** Correlation between density of immune cell infiltration at invasive margin (IM) and tumor core (TC) of CD45RO-TILs (n = 18, r = 0.68; p = 0.002 by Spearman analysis) (**e**) and CD8-TILs (**f**) (n = 18, r = 0.64; p = 0.004 by Spearman analysis)

By immunohistochemistry, we analyzed the infiltration of CD8+ , CD45RO+ , PD-1+ T cells (from now on referred to as tumor-infiltrating T cells, TILs), IL-17+ cells, CD20+ B cells and CD68+ tumor-associated macrophages (TAM) (Fig. 1b–h). Because CD20+ B cells localized not only as interspersed cells (Fig. 1f) but also as dense aggregates (Fig. 1g), we analyzed these two immune compartments as distinct, namely CD20-TILs and CD20-TLT [20]. In general, for all the immune types considered, immune cells localized both in the tumor core (TC) and at the invasive margin (IM). Notably, a considerable number of immune cells was also present in the mucosa of the peritumor healthy tissue, in particular CD8-TILs and CD68+ macrophages (Fig. 1c, h). Quantitative assessment by computer-assisted image analysis of immune cell densities (immunoreactive area (IRA%)) at the invasive margin of adenocarcinoma specimens revealed heterogenous distribution of the different cell types across patients, with median IRA% (IQR) respectively CD45RO-TILs 2.19 (2.14), CD8-TIL 0.42 (0.81), CD20-TILs 0.22 (0.51), CD20-TLT 2.84 (4.64), CD68-TAM 2.19 (1.56), IL17+ cells 0.39 (0.39), PD1-TILs 0.19 (0.41) (Fig. 2a). With the exclusion of CD20-TLT, for which we specifically analyzed those areas where they were localized (Fig. 1g), the most frequent immune type was by far CD45RO-TILs, closely followed by CD68-TAM, with a maximum value of 4.7 and 5.3 respectively.

As in other tumor types, B cells were preferentially located in tertiary lymphoid tissue (CD20-TLT) rather than interspersed (CD20-TILs), confirming previous findings [20]. Spearman correlation analysis showed a significant correlation among CD45RO-TILs and IL17+ cells (p = 0.007; r = 0.61) (Fig. 2b), whereas CD8-TILs correlated with PD1 TILs (p = 0.023; r = 0.53) (Fig. 2c). In contrast, CD20+ B cells correlated only among themselves (TLT and TILs) (p = 0.013, not shown), suggesting that they could represent an immune compartment distinct from the others analyzed. For the two lymphoid immune populations most relevant in prognostic studies performed in CRC, CD45RO-TILs and CD8-TILs, we analyzed whether there was a preferential localization at the invasive margin (IM) or tumor core (TC) and we found very similar values (Fig. 2d) and a significant concordance of the distribution in the two compartments (p = 0.002 and p = 0.004) (Fig. 2e, f).

### Variation of immune cell infiltration along the process of tumorigenesis and according to histotypes

**Fig. 3:**
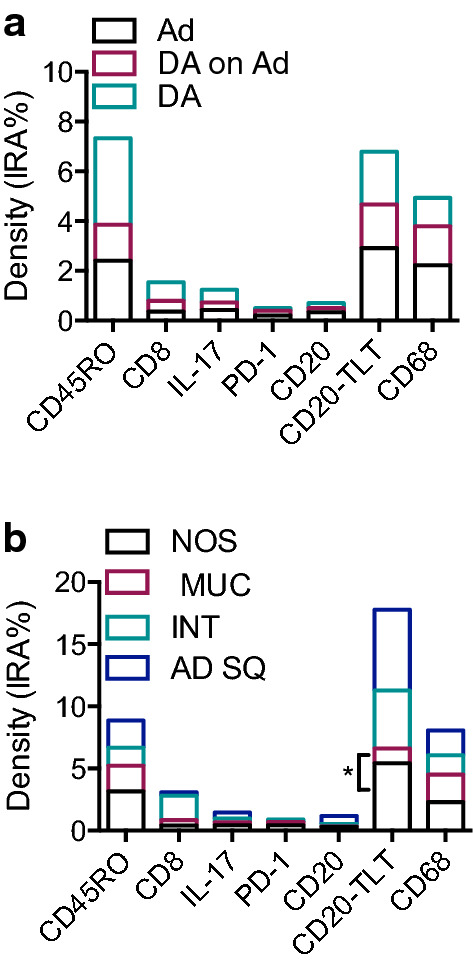
Distribution of immune populations according to histopathological features. **a** Immune cell density (IRA%) in DA (Duodenal Adenocarcinoma; n = 12); DA on Ad (Duodenal Adenocarcinoma progressed from Adenoma; n = 3) and Ad (Adenoma; n = 3). **b** Density of immune cells in duodenal cancer specimens according to histotype: NOS (Not Otherwise Specified; n = 7); MUC (Mucinous histotype; n = 5); INT (Intestinal histotype; n = 1); AD SQ (Adenosquamous histotype; n = 1). * p = 0.01 by Mann–Whitney

To test whether immune cell infiltration varies according to evolution of disease, we compared the density of immune cells in adenomas, adenocarcinomas and adenocarcinomas progressed from adenomas. Surprisingly, the distribution of immune cells in the three groups was quite homogeneous for all the immune variables considered (Fig. 3a), thus not evidencing a marked modification of immune infiltrate in malignant lesions compared to benign ones. Considering the histotype, CD20-TLT infiltrate was significantly lower in DA with a mucinous histotype compared to the NOS DA (median:1.173 vs median:5.437; p = 0.01 by Mann–Whitney) (Fig. 3b). Moreover, we overall observed a less abundant B and T lymphocyte infiltration in mucinous DA compared to NOS DA, even if not statistically significant, possibly because of the heterogeneity and scarce numerosity.

### Correlation of immune cells with prognostic factors and recurrence

**Fig. 4:**
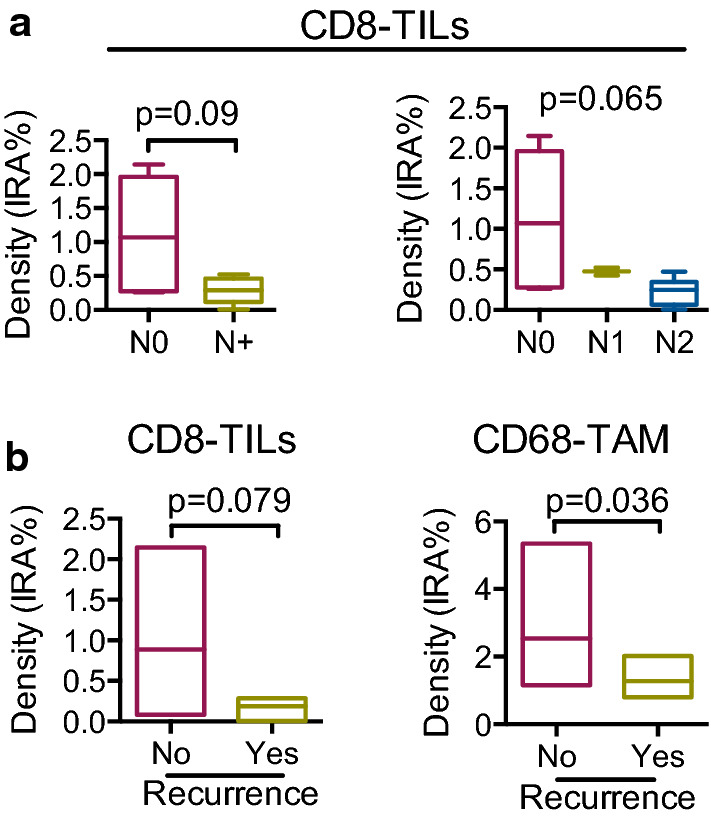
Correlation of immune cell density with clinical variables. **a**, **left** Density (IRA %) of CD8-TILs according to presence (N+ ; n = 8) or not (N0; n = 7) of metastatic lymphnodes (p = 0.093 by Mann–Whitney). **a**, **right** Density (IRA %) of CD8-TILs according to N stage (N0: no lymph nodes, n = 7; N1: 1 metastatic lymph node, n = 2; N2, more than 1 metastatic lymph node, n = 6; p = 0.064 by Kruskal–Wallis). **b** Density (IRA %) of CD8-TILs (**left**) in patients with recurrence (n = 3) and no recurrence (n = 9; p = 0.09 by Mann–Whitney) and density (IRA %) of CD68-TAMs (**right**) in patients with recurrence (n = 3) and no recurrence (n = 9; p = 0.036 by Mann–Whitney)

Then, we tested the relationship of immune cell infiltration with well-known clinical and pathological variables, commonly used as prognostic factors. At Spearman correlation analysis, we found a significant inverse correlation between infiltration of CD8-TILs and lymphnode ratio (LNR) (p = 0.013) and between density of CD8-TILs and number of metastatic lymphnodes (p = 0.019) (Table 2). Accordingly, density of CD8-TILs was lower in N+ adenocarcinomas relatively to N0, albeit not significantly (median 0.29 vs 1.07; p = 0.090 by Mann–Whitney), (Fig. 4a, left). Furthermore, stratifying patients for the N status, we observed a difference of CD8-TILs density among groups (p = 0.065 by Kruskall-Wallis) (Fig. 4a, right). The density of CD8-TILs progressively decreased with the increasing of the N stage, suggesting that CD8-TIL infiltration may be protective against nodal metastatization. We also observed a correlation between CD45RO-TILs and LNR, albeit not significant (p = 0.090), while no significant correlation was found between density of CD45RO-TILs and nodal metastatic status.

**Table 2:** Spearman’s Rho correlation between immune cells and lymphnode invasion

	LNR	n° LN+
CD45RO mean %area		
Correlation coefficient	− .452	− .369
Sig. (2-tailed)	.091	.176
CD8 mean % area		
Correlation coefficient	− .625(*)	− .596(*)
Sig. (2-tailed)	.013	.019
CD20 TIL mean %area		
Correlation coefficient	− .136	− .110
Sig. (2-tailed)	.630	.697
CD20 TLT mean %area		
Correlation coefficient	− .422	− .340
Sig. (2-tailed)	.117	.215
CD68 mean %area		
Correlation coefficient	− .463	− .361
Sig. (2-tailed)	.082	.186
IL-17 mean %area		
Correlation coefficient	− .429	− .390
Sig. (2-tailed)	.110	.150
PD-1 mean %area		
Correlation coefficient	− .177	− .125
Sig. (2-tailed)	.528	.657

*Correlation is significant at the 0.05 level (2-tailed)

** Correlation is significant at the 0.01 level (2-tailed)

LNR: Lymph node ratio. n° LN+ : number of metastatic lymph nodes

When looking at recurrence, Mann–Whitney analysis evidenced a lower density of CD8-TILs in patients who experienced recurrence and died for the disease, albeit not significant (median:0.276 vs 0.524; p = 0.079) (Fig. 4b, left). Notably, CD68-TAM distribution was significantly different in patients who had recurrence versus patients who did not (median 1.028 vs 2.276; p = 0.036) (Fig. 4b, right), suggesting a protective role also for macrophages, in line with data on human CRC. As to CD45RO-TILs, their density was not associated to recurrence (p = 0.165).

## Discussion

The aim of this study was to document density and type of immune cells in duodenal cancer, an extremely rare neoplastic condition, for which limited knowledge of the immune microenvironment is available. Our study encompassed the analysis of specific types of immune cells that we selected based on current literature. In particular, CD45RO+ , indicating memory T cells, CD8+ T cells, CD20+ B cells and CD68+ macrophages have been convincingly shown to associate with prognosis in the context of other gastrointestinal malignancies (i.e. colo-rectal cancer and pancreatic adenocarcinoma) [20–24], while IL-17 (indicating Th17 T cells mostly, but also other myeloid types) and PD-1 identify cells with critical roles at mucosal sites [25–27] or in the context of immunotherapy [28–30] respectively.

CD45RO+ cells were the most frequent immune type, closely followed by CD68-TAMs, while PD-1+ T cells and IL-17+ cells were found at lower levels compared to other immune types, as expected. Besides the density of immune cells, we considered their localization either in the core or at the invasive margin of the tumor and the presence of scattered cells versus the organization in lymphoid aggregates, in order to adequately capture critical features of the immune microenvironment. According to localization, CD45RO+ T cells and CD8+ T cells, the key variables considered for the Immunoscore in colo-rectal cancer (CRC), presented no major difference, both the populations being uniformly distributed at the invasive margin and in the tumor core. According to distribution, infiltrating CD20+ B cells were preferentially localized in lymphoid aggregates, although there was a correlation between the density of CD20-TILs and CD20-TLT.

Distribution of immune cells in adenomas compared to duodenal cancers or duodenal cancers progressed from adenomas was quite homogeneous, suggesting a lack of modification of immune infiltrate in malignant and benign lesions. This could be ascribed to different reasons; in the first place, the presence of immune cells in benign lesions (adenomas) could mirror an early activation of the immune response in the early steps of tumorigenesis. At the same time, however, it should be pointed out that the mucosa of the small bowel hosts resident populations of immune cells that could interfere with our analysis focused on tumor-infiltrating cells. Since we do not have any means to discriminate resident from infiltrating immune cells, this hypothesis remains open and only future analyses encompassing control specimens could help answering this question.

In human pancreatic adenocarcinoma (PDAC), density of B cells within lymphoid tissue (CD20-TLT) was significantly associated with longer survival [20]. A possible explanation, substantiated by evidence in the literature and gene expression signature of B cells, is that B cells in lymphoid tissue receive important activation signals that can be critical to mount an antitumor immune response. Lymphoid tissue has been associated to favourable prognosis also in colo-rectal cancer patients [22]. In contrast, no prognostic function or association with clinical features was observed for CD20+ B cells in duodenal cancer. This could be due to the fact that lymphoid tissue is a common component of the gut-associated lymphoid tissue (GALT) and neoformation of lymphoid tissue, as observed in PDAC, does not confer a benefit during cancer progression.

A major point of our study was to assess the clinical relevance of immune cells in DA, by testing the relationship of immune cell infiltration with well-known clinical-pathological prognostic factors. We found a significant inverse correlation of CD8+ cell density with nodal involvement, suggesting that an increased number of these cells could protect from nodal metastasis. This was further corroborated by a decreased CD8-TIL infiltration in patients who relapsed suggesting that adaptive immune escape and particularly lack of cytotoxic T cell infiltration may correlate with a more aggressive phenotype, as is the case in CRC [14]. As to CD45RO, commonly used in prognostication studies, this marker was not significantly associated to clinical variables in duodenal cancer. Possible explanation could be that expression of this marker is not capable to capture specific immune subsets with a defined antitumor fucntion. The positive prognostic value of CD8-TILs may seem in contrast with the correlation between CD8 and PD-1, since PD-1 is usually a marker of exhausted lymphocytes. However, PD-1+ T cells have multiple functions, far from being merely associated to immunosuppression [31]. More detailed studies are required to define the profile of T cells infiltrating DA. There was also a significant correlation of CD68+ TAMs with absence of recurrence, again in line with studies showing a positive prognostic function of macrophages in human CRC [24, 32]. Interaction of macrophages with anticancer treatments, including chemotherapy, has been shown to polarize their function towards an antitumor mode; this possibility remains to be tested in DA.

## Conclusions

Our analysis provides a novel characterization of immune infiltrate in DA. We describe how different immune subsets are related to well-known clinical and pathological characteristics of the disease, and suggest that T cell infiltration may play a protective role towards lymphatic spread of disease and nodal metastatization. Also, T cell density and macrophage infiltration may lower the risk of recurrence and disease related death. Despite the fact that our findings are in line with results of previous studies conducted in other malignancies including CRC and pancreatic cancer, a clear limitation of our study was the limited number of specimens determined by the low incidence of the disease. A multicentric approach, allowing analysis of larger cohorts of patients, could circumvent this constraint and increase the power of our observations. It would also be useful to investigate a larger number of adenomas, possibly including endoscopically resected lesions, in order to better characterize the evolution of immune infiltrate along the process of tumorigenesis. Ultimate goal is including easily assessable immune markers in the prognostic stratification algorithm of duodenal cancer, to better guide post-operative treatment.

## Data Availability

All materials and data are stored at Humanitas Clinical and Research Center and may be shared upon request directed to the corresponding authors.

## Abbreviations

DA: Duodenal adenocarcinoma
Ad: Adenoma
SBC: Small bowel cancer
TNM: Tumor-Nodes-Metastasis staging system
N: Lymphnode
TME: Tumor microenvironment
CRC: Colorectal cancer
PDAC: Pancreatic ductal adenocarcinoma
TC: Tumor core
IM: Invasive margin
CT: Computed tomography
EGDS: Esofago-gastro-duodenoscopy
EUS: Endoscopic ultrasound
FNAB: Fine needle aspiration biopsy
EDTA: Ethylenediaminetetraacetic acid
IQR: Interquartile range
RFS: Recurrence-free survival
DSS: Disease-specific survival
IRA: Immune reactive area
NOS: Not otherwise specified
TILs: Tumor infiltrating lymphocytes
TLT: Tertiary lymphoid tissue
TAM: Tumor associated macrophages
